# Effect of Poly(vinyl alcohol) Concentration on the Micro/Mesopore Structure of SBA15 Silica

**DOI:** 10.3390/ma15248900

**Published:** 2022-12-13

**Authors:** Seongmin Kim, Minuk Jung, Seongsoo Han, Ho-Seok Jeon, Yosep Han

**Affiliations:** 1 Mineral Processing & Metallurgy Research Center, Resources Utilization Division, Korea Institute of Geoscience & Mineral Resources (KIGAM), 124, Gwahak-ro, Yuseong-gu, Daejeon 34132, Republic of Korea; 2 Department of Resources Recycling, University of Science and Technology (UST), 217, Gajeong-ro, Yuseong-gu, Daejeon 34113, Republic of Korea

**Keywords:** self-assembly, P123 micelles, PVA, SBA15, pore structure

## Abstract

In this work, a series of micro/mesoporous SBA15 silica materials were synthesized using P123 and poly(vinyl alcohol) (PVA) as co-templates. The pore structure of the prepared SBA15 was observed to be a function of the PVA concentration. When the amount of PVA was relatively small, the specific surface area, micropore volume, and pore wall thickness of the synthesized SBA15 were considerably large. By contrast, when a large amount of PVA was added, the pore wall thickness was greatly reduced, but the mesopore volume and size increased. This is because the added PVA interacted with the polyethylene oxide (PEO) in the shells of the P123 micelles. Furthermore, when the amount of PVA was increased, the core polypropylene oxide (PPO) block also increased, owing to the enhanced aggregation of the P123/PVA mixed micelles. This research contributes to a basic comprehension of the cooperative interactions and formation process underlying porous silica materials, assisting in the rational design and synthesis of micro/mesoporous materials.

## 1. Introduction

Nanoporous silica with a 2–50 nanometers pore diameter is a porous material that consists of regularly arranged pores [1,2,3,4,5,6,7,8,9,10,11,12,13,14,15,16,17,18,19,20]. It has been prevalently employed in catalyst supports, drug delivery carriers, adsorbents, sensors, and fuel cells, owing to its high specific surface area, uniform pore structure, narrow pore size distribution, and potential ability to inhibit active particle growth and aggregation in its pores [1,2,3,4,5,6,7,8,9,10,11,12,13,14,15,16,17,18,19]. In addition, porous materials such as zeolite and MOFs have been researched, and mesoporous silica has many advantages which enable it to be used in various applications, such as high diffusivity using relatively large mesopores and high thermal/chemical stability [1,2,3,4,5,6,7,8,9,10,11,12,13,14,15,16,17,18,19,20,21,22,23]. Several types of mesoporous materials (e.g., SBA, MCM, KIT, etc.) have been manufactured by modifying the synthesis pathway and surfactants [1,2,3,4,5,6,7,8,9,10,11,12,13,14,15,16,20,21]. Due to its thick pore wall and relatively high hydrothermal stability, SBA15 has received the most attention among these mesoporous materials, and various techniques have also been used to further increase its hydrothermal stability [1,2,3,4,5].

The pore characteristics of mesoporous silica depend on the synthesis procedures, and these can vary considerably. The characteristics relevant to the aforementioned applications are the pore geometry, diameter, size distribution, volume, and BET surface area. Numerous research groups have tailored the pore structure of mesoporous materials to optimize their properties [1,5,6,7,8,9,10,11,13,14,15,17,24,25,26,27,28]. To enhance the applicability of these materials, it is necessary to increase their pore volume and BET surface area.

Zhong et al. prepared a micro/mesoporous composite, SBA15, as a catalyst support. Different amounts of copper were added to the mesoporous silica to yield materials with different pore diameters and micro/mesopore fractions [15]. Colilla et al. prepared SBA15 via a templated sol-gel synthesis by using HCl as a catalyst and adding different amounts of phosphoric acid to the precursor mixture [20]. The synthesized SBA15 possessed improved surface properties, and it is an excellent candidate material for many biomedical applications. Zhu et al. proposed that the addition of polyvinyl chloride (PVA) during SBA15 synthesis enhances the surface area and mesophase porosity of the obtained catalyst support without interfering with its synthesis [25]. Erdem et al. investigated the effect of polyethylene glycol (PEG) on the catalytic activity of SBA15 in the esterification of propionic acid with methanol [9]. They concluded the addition of PEG increases the surface area of the mesochannels and promotes mass transfer in addition to allowing the generation of a stable mesoporous replica.

In this study, mesoporous SBA15 silica was synthesized using P123 as a template and an appropriate amount of the hydrophilic polymer PVA as an auxiliary templating agent. The pore and structural characteristics of the synthesized SBA15 were investigated through nitrogen adsorption using the Barrett–Joyner–Halenda (BJH) and non-local density functional theory (NLDFT) models. These results indicated that the added PVA concentration affects the pore structures of the SBA15 such as the pore size and volume and the specific surface area et al. Furthermore, the role of PVA in the preparation of SBA15 with Pluronic P123 as a template was studied using a quartz crystal microbalance with dissipation monitoring (QCM-D) and dynamic light scattering (DLS) particle-size-distribution analysis.

## 2. Materials and Methods

### 2.1. Materials and Chemicals

All of the chemical compounds were used as received without further purification. The triblock copolymer surfactant, polyethylene oxide-*block*-polypropylene oxide-*block*-polyethylene oxide (P123, EO_20_PO_70_EO_20_, M_w_ = 5800, 30 wt%), silicate precursor, tetraethylorthosilicate (TEOS, 98%), hydrochloric acid (HCl, 37%), and polyvinyl alcohol (PVA, M_w_ = 130,000) were obtained from Sigma-Aldrich (St. Louis, MO, USA). An aqueous PVA solution (1 wt%) was manufactured by dissolving the PVA in 90 °C water.

### 2.2. Synthesis of Mesoporous SBA15 with PVA Addition

Mesoporous SBA15 samples were synthesized under the conditions that are described in the literature [3,16,29]. The 4.0 g of P123 was dissolved in 120 g of 2 M HCl and 30 g of DI at 35 °C. After P123 was fully dissolved, a freshly prepared aqueous PVA solution (1 wt%) was added at different concentrations. Then, 8.5 g of TEOS was added to the solution, stirred at 35 °C for 1 d and aged at 90 °C for 1 d. The sample was obtained by filtration, drying and heating it in a furnace at 550 °C for 4 h to remove the surfactant. The synthesized SBA15 was represented by SBA15-A-X for each added PVA concentration (x = 0.75, 3, 5 and 10).

### 2.3. Characterization

Small-angle X-ray diffraction (SAXRD) patterns were measured using a Bruker D8 HRXRD X-ray diffractometer with Ni-filtered Cu Kα radiation (λ = 0.154606 nm, 40 kV, 40 mV) and Nanostar U small-angle X-ray scattering from 0.5° to 4.0° (2θ) with a resolution of 0.001°. A physisorption analyzer (3Flex, Micromeritics, Norcross, GA, USA) was used to measure the isotherms of the nitrogen (N_2_) adsorption and desorption at the liquid nitrogen temperature. The Brunauer–Emmett–Teller (BET) method was then used to analyze the specific surface area for a relative pressure (P/P_O_) range of 0.05–0.25 [24,30]. The N_2_ adsorption branch was used to determine the mesopore volume and size distribution, and the BJH method was used to determine the mesopore size distribution [24,31,32]. In addition, the micropore volumes (i.e., intra-wall pores) of the samples were obtained from the adsorption branch based on the NLDFT model with the assumption that the pores are cylindrical [24,33]. To observe the microstructure and morphology of the synthesized samples, transmission electron microscopy (TEM) was performed using a JEOL-2100F operated at 200 kV.

### 2.4. Variation in P123 Micelle Properties with PVA Concentration

In addition to monitoring the dissipation, the measurements were made using a quartz crystal microbalance (QCM-D; QSence, Biolin Scientific, Gothenburg, Sweden). The silica sensors were treated in an ultraviolet (UV)-ozone chamber for 30 min before the measurement by being submerged in 2% sodium dodecyl sulfate, washed with DI, filtered and dried with Ar gas. Pure P123 micelles and mixed P123/PVA micelles were continuously pumped through the chambers during all of the measurements, which were conducted at 90 °C. An intrusion pump was used at 50 μL/min. The QCM-D analyzer measures the fundamental frequency of the sensor crystal allowing simultaneous determination of *f* and energy dissipation *D*. Depositing a uniform layer of mass Δm in the crystal reduces the resonant frequency by Δf with the Sauerbrey relation [34,35]:

(1) Δm=−(C/N)Δf 

where *N* is the number of overtones and *C* is the integrated crystal sensitivity that depends on the intrinsic properties and thickness of the crystal. The voltage above the crystal decreases exponentially when the driving power to the piezoelectric oscillator is switched off and the damped oscillating signal is memorized, providing the energy dissipation measurement. The following is a definition of the dissipation factor:

(2) $$D=E_{d}/2\pi E_{s}$$

where $E_{d}$ is the energy dissipated during one oscillation, and $E_{s}$ is the energy stored in the oscillating system [36].

The size distribution of the micelles was measured by dynamic laser scattering (DLS) using an ELS-Z. The P123 micelle size by the addition of PVA solution was determined under the same conditions as the blank solution condition (e.g., 90 °C) when we were synthesizing SBA15.

## 3. Results and Discussion

### 3.1. Mesopore Structure of Mesoporous SBA15 with PVA Addition

**Figure 1:**
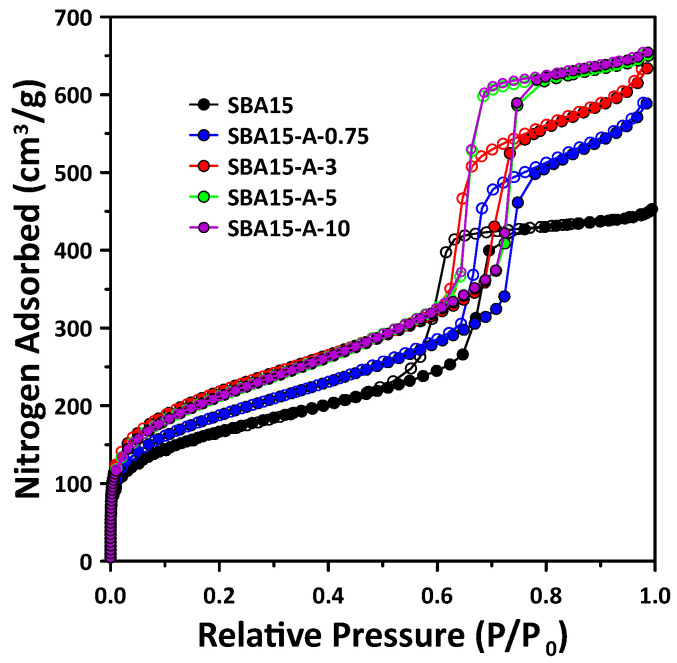
Nitrogen adsorption-desorption isotherms of the SBA15 synthesized using different concentrations of PVA solution compared with that of pure SBA15. The solid and empty circles display the adsorption and desorption curves, respectively.

**Figure 2:**
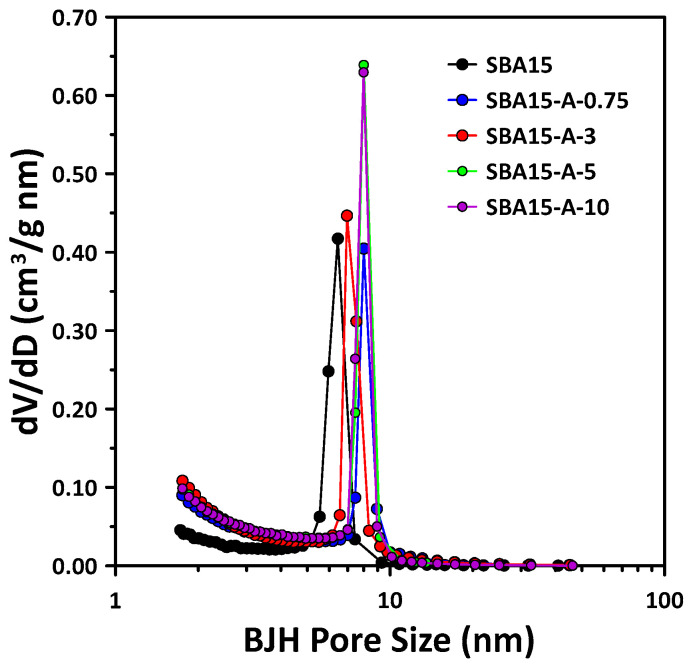
Mesopore size distribution particle size of the SBA15 synthesized with different concentrations of PVA compared with that of pure SBA15. Mesopore size distributions calculated using BJH method from the N_2_-adsorption isotherms are shown in Figure 1.

Figure 1 and Figure 2 show the N_2_ ad-desorption isotherms and mesopore size distribution of the SBA15 samples fabricated with various amounts of PVA. As shown in Figure 1, all of the samples show type IV isotherms with an H1 hysteresis loop according to the IUPAC classification, suggesting the formation of cylindrical and hexagonal mesoporous structures using P123 as a surfactant [3,16,24]. In addition, as the amount of adsorbed nitrogen and hysteresis point (P/P_0_) of the sample synthesized with PVA increased, the pore volume, specific surface area and pore size also increased. It can be confirmed that the surface area and pore volume increased owing to the formation of additional pores with small diameters in the synthesized SBA15. These pores formed as a result of the interaction of micelles generated from P123 with PVA during the SBA15 synthesis.

As shown in Figure 2, a uniform pore size of ≈6–8 nm was obtained, and the pore diameter increased slightly with the addition of PVA. Therefore, the PVA concentration had a small effect on the pore diameter in the synthesized SBA15. In particular, since the size of the mesopores is known to be mainly affected by the PPO block contained within the templating agent P123, PVA may not have fully reacted with the PPO block.

### 3.2. Micropore Structure of Mesoporous SBA15 with Addition of PVA

**Figure 3:**
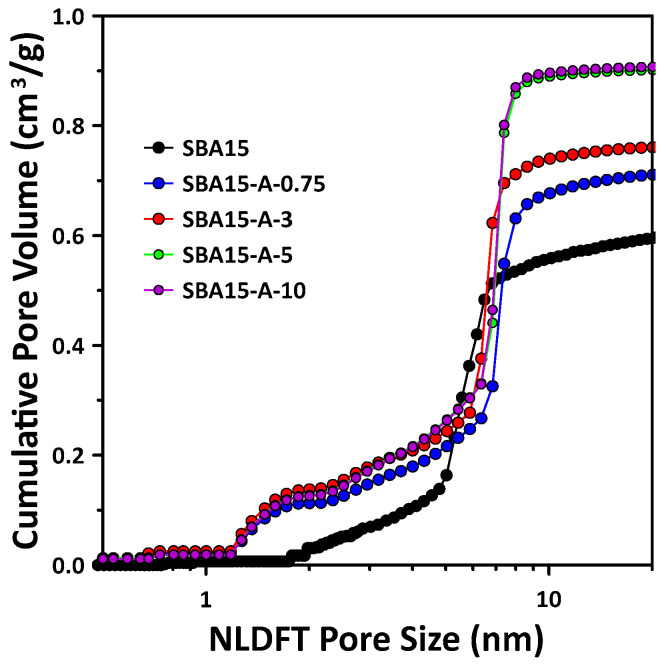
Cumulative pore volume of SBA15 synthesized with different PVA concentrations compared with that of pure SBA15 as determined using NLDFT method. Pore size distributions derived from the N_2_-adsorption isotherm are shown in Figure 1.

From the previous results (isotherms and BJH mesopore size distribution), it is concluded that the N_2_ adsorption amount and pore size slightly increased owing to the addition of PVA. The triblock copolymer (i.e., P123, PEO-PPO-PEO block) used in the hydrothermal synthesis of SBA15 is generally known to induce the formation of micelles with hydrophilic PEO as a shell and hydrophobic PPO as a core [9]. Here, the mesopores were formed from the PPO block, and the micropores were created from the PEO chain in the mesopore wall of the final synthesized SBA15 sample; therefore, the micropore volume increased as the thickness of the pore wall increased [12,18]. We observed the change in the micropores owing to the addition of hydrophilic PVA during the synthesis of SBA15, and the results are shown in Figure 3.

In general, the micropores formed in the mesopore wall of the SBA15 material are assumed to be cylindrical to determine the micropore size distribution. Therefore, in this study, the micropores of all of the synthesized samples were assumed to be cylindrical, and the NLDFT model was used to calculate the micropore size distribution. Figure 3 shows the results for the SBA15 synthesized with different PVA concentrations and pure SBA15 (no added PVA). Notably, in contrast to pure SBA15, all of the samples synthesized with PVA possessed abundant, large micropores (<2 nm). In particular, the number of pores in the size range of ≈0.3–2.5 nm was considerably increased owing to the addition of PVA. This finding confirmed that the addition of PVA in the SBA15 synthesis process induced micropore formation, owing to the effect of P123. Hydrophilic PVA is assumed to have an effect, such as interacting with the PEO chains, which act as a shell in P123. Therefore, we can expect that the mesopore wall thickness will increase if the micropore size increases as a result of the interaction with the PEO chain.

To confirm the PVA effect on pore structure of all of the prepared samples, Table 1 presents the textural properties (e.g., surface area, micro/mesopore size, volume and wall thickness) of the samples synthesized with PVA and those of pure SBA15. As shown in Table 1, the Bragg peak (100) was determined to be 9.25 nm for SBA15, and it was 10.62, 10.56, 10.47 and 10.43 nm for SBA15-A-0.75, 3, 5 and 10, respectively. Overall, the pore spacing (100) peak for SBA15 synthesized with PVA increased compared with that of pure SBA15. According to the previous result, if the mesopore size of the samples are the same, it is known that an increase in the pore spacing naturally means that there is an increase in the mesopore wall thickness [18,24]. Notably, Table 1 shows the surface areas of the SBA15 synthesized with PVA, which are higher than that of pure SBA15. SBA15-A-3 had the highest specific surface area (807.3 m^2^/g), but the samples synthesized with higher concentrations of PVA had slightly lower specific surface areas (SBA15-A-5 = 775.1 m^2^/g; SBA-15-A-10 = 772.7 m^2^/g).

Moreover, the micropore volume calculated using the NLDFT method also increased significantly in the PVA-added samples compared with that of pure SBA15, and SBA15-A-3 had the highest micropore volume. The micropores in the mesoporous SBA15 silica are formed on the mesopore wall by the PEO chain of the P123 surfactant [37]. Previous studies have attributed the increase in the BET surface area of the SBA15 sample to the micropores, which improves the mesopore connectivity [18]. Therefore, as the micropores are formed in the mesoporous wall, the increased wall thickness may account for the increase in the BET surface area of the mesoporous SBA15 silica [24,38]. In this study, the wall thickness also increased with an increase in the surface area and micropore volume, owing to the addition of PVA. In particular, the wall thickness increased maximally in SBA15-A-3, which had a high specific surface area and micropore volume. The increase in the specific surface area and micropore volume upon the PVA addition is probably due to the PVA-induced increase in the mesopore wall thickness and micropore formation. These results demonstrate that the interactions entailing adsorption or adhesion on the shell PEO chains of P123 micelles likely arise from the addition of a hydrophilic PVA polymer.

However, upon the addition of a relatively high concentration of PVA (SBA15-A-5 and SBA15-A-10), the wall thickness of the mesopores was significantly reduced compared to that of SBA15-A-3. Specifically, the mesopore wall thickness of SBA15-A-5 and SBA15-A-10 was not significantly greater than that of the pure SBA15. Notably, as the concentration of PVA increased, the mesopore size increased slightly compared with that of the pure SBA15, but it subsequently remained constant. In addition, the mesopore volume increased as the concentration of PVA increased, and thus, the total pore volume (micro/mesopore volume) also increased. As a result, it was confirmed that the P123 micelles were affected by the additional amount of hydrophilic PVA present during the SBA15 self-assembly process. Therefore, we characterized the interactions (i.e., adsorption and adhesion) and size distribution of the P123 micelles according to PVA concentration and analyzed the underlying mechanism.

### 3.3. Microstructure and Morphology of SBA15 Synthesized with PVA

**Figure 4:**
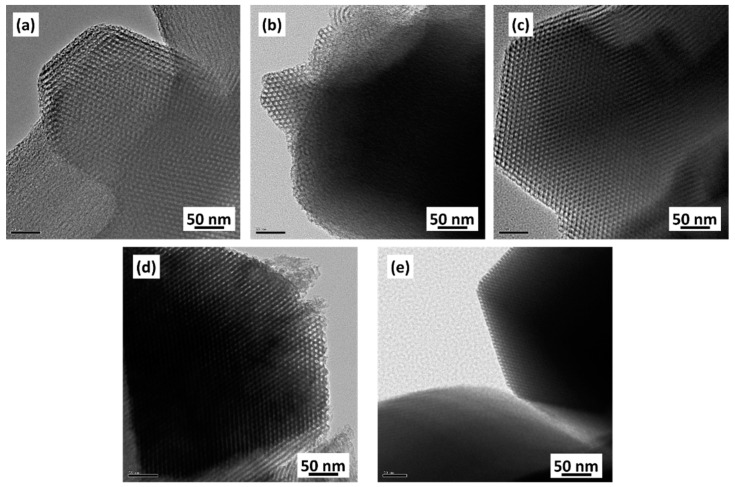
TEM images of the SBA15 samples synthesized with different PVA addition concentrations: (**a**) SBA15 (no added PVA), (**b**) SBA15-A-0.75, (**c**) SBA15-A-3, (**d**) SBA15-A-5 and (**e**) SBA15-A-10.

To observe the microstructure and morphology of SBA15 fabricated with various concentrations of PVA, the samples were analyzed using TEM (Figure 4). According to the TEM images, all of the samples possessed well-ordered hexagonal arrays of mesopores, regardless of the PVA concentration. The pore size was 6–8 nm, and the distance between the adjacent pores was 9–10 nm, which is in agreement with the N_2_ ad/de-sorption isotherms and the unit cell parameters calculated from XRD.

### 3.4. Self-Assembly Properties of P123/PVA Mixed Micelles According to PVA Concentration

To confirm that the change in the micro/mesoporous structure of the synthesized SBA15 depends on the PVA concentration, we analyzed the adsorption characteristics and size distribution resulting from the interaction between the P123 micelles and PVA during the synthesis process. The changes in the P123 micelles after the PVA addition were observed using the QCM-D and DLS analyses.

**Figure 5:**
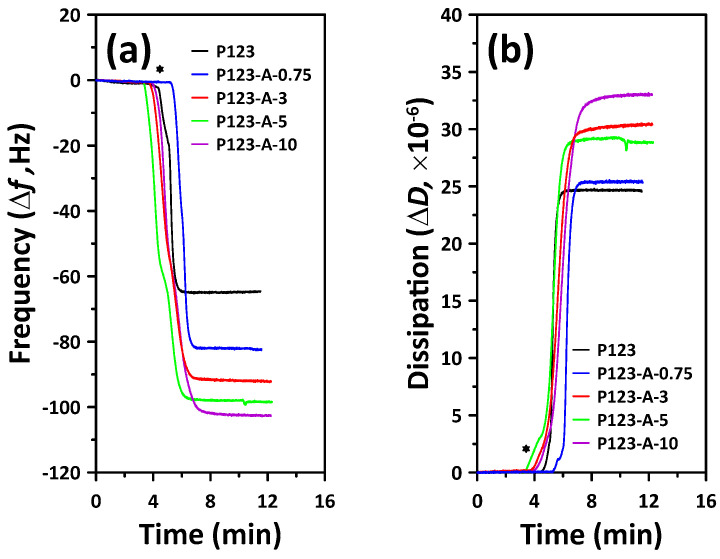
QCM−D trace for P123 micelles in aqueous PVA solutions of different concentrations at 90 °C. The traces show changes in both (**a**) frequency and (**b**) dissipation for the third harmonic (*n* = 3 and f = 15 MHz) of a given experimental flow over the SiO_2_ surface. The asterisks (*) indicate the point at which the P123 copolymer solutions containing different concentrations of PVA flowed into the chamber.

In order to obtain results regarding the adsorbed material mass in a time-resolved manner, QCM-D detects the quartz crystals resonance frequency (f) [35,36]. The physical characteristics of the P123/PVA mixed micelles can be ascertained by estimating the acoustic parameters such as the dissipation factor (D) and resonance frequency, as well as the viscoelasticity and thickness of the adsorbed layer attached to the sensor surface [35,36]. Figure 5 shows the profile traces for the pure P123 micelles and P123/PVA complex micelles that were formed during the synthesis with different PVA concentrations at 90 °C. In all of the samples, the interaction with the hydrophilic SiO_2_ surface manifested as a rapid frequency change upon the chamber injection procedure. Based on these results, we believe that the P123 micelles formed under the SBA15 synthesis condition are not torn or ruptured on the hydrophilic silica surface, but they are deposited as a triblock PEO-PPO-PEO core–shell structure.

Notably, the P123/PVA mixed micelles exhibited a higher Δf than the pure P123 micelles did. Moreover, the variation in Δf was analyzed with respect to the amount of PVA, where the largest Δf among the mixed micelles was observed for P123-A-10. Based on these data, viscoelastic modeling was conducted using the Voigt model, and the results are listed in Table 2. We ensured reliability by conducting at least 10 experiments, and all of the results are represented by the means and standard deviations. In the low-PVA-concentration range (PVA-0.75 and PVA-3), the layer thickness increased only slightly compared with that of the pure P123 micelles. In addition, the areal mass output of the model significantly increased. Thus, it is clear that the PEO shell of P123 was affected in the cases of PVA-3 or lower, and as a result, the highest wall thickness and micropore volume were observed in the final product with PVA-3 (SBA15-A-3).

**Figure 6:**
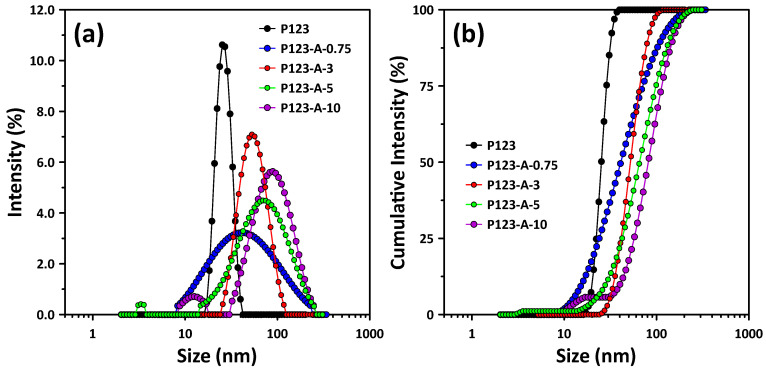
DLS-determined size distributions of the P123/PVA micelles compared with that of the P123 aqueous solution: (**a**) intensity distributions and (**b**) cumulative intensity curves.

However, it was observed that the layer thickness and areal mass increased significantly at relatively high PVA concentrations (PVA-5 and PVA-10). In SBA15-A-5 and SBA15-A-10, the wall thickness decreased and the mesopore volume increased significantly after the addition of PVA. Therefore, when the amount of PVA added was excessive, the interaction of PVA with the P123 core–shell structure would not be sufficiently explained by the results of the QCM-D experiment alone. To elucidate this finding, using DLS, we observed the change in the P123 micelles as the PVA concentration was varied; the results are listed in Figure 6.

Figure 6 presents the effect of the PVA amount on the aggregation behavior of P123 in the solutions at 90 °C. The mean hydrodynamic diameter (d_50_, D_h_) of the pure P123 was 25.3 nm. Further, the micelle size distribution shifted rightward as the concentration of PVA increased (the D_h_ of P123-A-0.75, P123-A-3, P123-A-5 and P123-A-10 was 40.6, 51.2, 65.3 and 80.7 nm, respectively), resulting in the formation of micellar aggregates and an increase in the aggregate size. Figure 6a shows only one peak for pure P123 and a low PVA concentration (≤PVA-3), but a relatively small secondary peak appeared at high PVA concentrations (≥PVA-5). In addition, fine nanoaggregates of 8.5–16.8 nm are evident in the curves of P123-A-10 with the highest addition amount. Since this size range is not observed in pure P123, the fine aggregates probably generated as a result of the addition of a large amount of PVA [37].

Notably, P123-A-3 had the best size uniformity among the P123/PVA mixed micellar aggregates (Figure 6b). Therefore, considering the above results, it is clear that the hydrophilic PVA interacts with the PEO shell. However, the addition of a relatively large amount of PVA increases the size of the P123/PVA mixed micellar aggregates, and eventually, the core PPO also exhibits a slight increase in size.

These results demonstrate that the mesopore connectivity of the SBA15 silica synthesized using P123 improved because of the formation of a large number of micropores upon the PVA addition. This improvement was the greatest for P123-A-3.

## 4. Conclusions

In this study, various concentrations of aqueous PVA solutions were added during a typical mesoporous SBA15 silica synthesis, and the micro/mesoporous structures of the synthesized samples were evaluated. As a result, significant changes in the micropore volume, mesopore wall thickness and mesopore volume were observed depending on the amount of PVA added. The results can be summarized as follows:

(1) The pore structure of SBA15 changed according to the added PVA concentration. In particular, increases in micropore volume and wall thickness were observed with the addition of PVA. SBA15-A-3 was confirmed to have the highest BET surface area and micropore volume, as well as the thickest pore walls. Notably, as the PVA concentration increased, the mesopore volume and size increased, resulting in a significant increase in the total pore volume.
(2) All of the SBA15 samples synthesized with PVA had a cylindrical and hexagonal mesopore structure, which is the typical pore shape of SBA15. The mesopore structure and morphology of the synthesized materials did not change with the PVA addition.
(3) At a certain PVA concentration (below P123-A-3), the PVA was able to interact with the shell PEO chains of P123 micelles, resulting in an improved micropore volume and specific surface area of the synthesized SBA15. However, the core PPO block also became larger owing to the aggregation of P123/PVA mixed micelles at high PVA concentrations.

Based on these experimental results, it was confirmed that the addition of hydrophilic PVA affects the formation of micropores and mesopores. The factors influencing the pore structure were determined based on the changes in the P123/PVA mixed micelles with the PVA concentration. This result confirmed the change of the P123 micelles acting as a template forming pores during SBA15 synthesis using the QCM-D and DLS analysis. In the future, it is expected that novel materials with hierarchical porous structures will be synthesized using this simple approach, in which various surfactants can be added (e.g., PEG). 

## Figures and Tables

**Table 1:** Textural properties of the SBA15 silica synthesized with different PVA concentrations.

Sample	d_100_ (nm) ^1^	Surface Area (m^2^/g) ^2^	Micropore Volume (cm^3^/g) ^3^	Mesopore Volume (cm^3^/g) ^4^	Mesopore Size (nm) ^4^	Total Pore Volume (cm^3^/g)	Wall Thickness (nm) ^5^
SBA15	9.25	599.8	0.031	0.541	6.65	0.596	4.03
SBA15-A-0.75	10.62	683.5	0.113	0.598	7.86	0.711	4.16
SBA15-A-3	10.56	807.3	0.137	0.624	7.48	0.761	4.82
SBA15-A-5	10.47	775.1	0.127	0.774	7.89	0.901	4.20
SBA15-A-10	10.43	772.7	0.125	0.782	7.91	0.907	4.13

^1^ d_100_ spacing measured using Bragg’s law from SAXRD; ^2^ Using BET model; ^3^ Calculated micropore (≤2 nm) volume from the adsorption isotherm based on the NLDFT method; ^4^ Determined using the BJH method based on the adsorption isotherm. The value for the N_2_ adsorption branch was calculated using a P/P_O_ of 0.3–0.8. The pore diameter, which can be evaluated using the calculated value, ranged from 2.0 to 50 nm. ^5^ Calculated using the $a_{O}$-BJH pore size ($a_{O}=2d_{100}/\sqrt{3})$.

**Table 2:** QCM-D data showing changes in the frequency and dissipation of the SiO_2_ surface in the presence of P123 surfactant and various concentrations of PVA ^1^.

Micelle Conditions	Δf (Hz)	ΔD $\left(\times 10^{-6}\right)$	Layer Thickness (nm)	Areal Mass $\left(\times 10^{11}\;\mathrm{ng}/{\mathrm{cm}}^{2}\right)$
P123	–64.68 ± 0.08	24.63 ± 0.04	38.4 ± 0.11	3.88 ± 0.01
P123-A-0.75	–82.45 ± 0.09	25.40 ± 0.05	38.7 ± 0.47	4.84 ± 0.06
P123-A-3	–92.16 ± 0.12	30.43 ± 0.05	39.6 ± 1.01	5.13 ± 0.11
P123-A-5	–98.44 ± 0.11	28.84 ± 0.03	101.2 ± 1.95	13.30 ± 0.24
P123-A-10	–102.57 ± 0.11	33.03 ± 0.06	98.6 ± 2.81	12.33 ± 0.35

^1^ Experiments were performed at least 10 times.

## Data Availability

Not applicable.
